# Miracles in medicine: A narrative inquiry exploring extraordinary events in pediatrics

**DOI:** 10.1002/hsr2.1623

**Published:** 2023-11-08

**Authors:** Geraldine Huynh, Marghalara Rashid, Jessica L. Foulds

**Affiliations:** ^1^ Department of Pediatrics University of Alberta Edmonton Canada

**Keywords:** miracles, narrative inquiry, palliative care, role of the physician, spirituality

## Abstract

**Background and Aims:**

Whether miracles are seen as a source of conflict between healthcare teams and families, a foolish or even harmful belief, or a hindrance to system‐wide improvements and scientific inquiry, miracles within medicine have long been questioned. We were interested in the perspectives of pediatricians on miracles and how that has informed their care of patients. We also sought to explore the intersection and relationship between the medical sciences, faith, and how we view miracles.

**Methods:**

Using narrative inquiry as a research methodology, we had conversations and explored the experiences of physicians who work directly within pediatric clinical care. We used purposeful sampling by emailing several pediatricians whom we felt might have been interested in the project and may have had experiences with perceived medical miracles. All conversations were conducted over Zoom and recorded.

**Results:**

We conducted one to three conversations with each participant. During our analysis, we identified two threads. First, participants described *miracles* mostly as favorable and fortuitous events that invoked a sense of wonder due to contextual factors such as geography, access to resources, time in history but also previous experiences and the expectations of the clinician. Second, there is a complex dynamic within and between the medical sciences and the faith commitments of clinicians and families, especially when facing life‐limiting or end‐of‐life illness.

**Conclusions:**

The stories that our participants told not only informed their past experiences and how they remembered them but also their present and future experiences. Exploring the topic of medical miracles allowed us to better understand the social discourses that shape our perceptions of miracles, death, and the role of the physician. These stories offer us hope and possibility in a time when we as a medical community may have lost our sense of wonder and the ability to notice miracles.

## 1 INTRODUCTION

On a cold January morning in 2007, a busker wearing a baseball cap, jeans, and a blue sweatshirt was playing violin for change in the L'Enfant Plaza Metro station in Washington, DC. A total of 1070 people passed the violinist without stopping or paying attention. According to *The Washington Post*, which later published an article on this famous social experiment, only two people stopped to take notice of world‐renowned musician, Joshua Bell, and his $3 million *Gibson Stradivarius*.^1^

The word *miracle* is derived from the Latin word *miraculum*, meaning a wonderful thing, a wonder, or marvel and is also related to the verb *mirari—*to wonder, be astonished, or amazed.^1^ Webster's 1913 dictionary defines a miracle as “an event contrary to the established course of things.”^2^ Miracles in medicine and the elite violinist at the metro station are both extraordinary occurrences that defy the expected course of events. Like the thousand commuters who overlooked the sweeping concertos of Joshua Bell and his violin, have we, as a medical community, lost our sense of wonder and the ability to hope for and even notice miracles?

Darshak Sanghavi labeled Alexander Fleming's discovery of penicillin and Edward Jenner's invention of the smallpox vaccine as miraculous events.^1^ He noted that these narratives of medical miracles or scientific breakthroughs tend to fit a specific motif—exciting “eureka moments” for the discoverer. The focus on sudden cures or revelations, he argued, can take away from the painstaking steps that are required in most medical advancements. Much can be said for the trials and errors, learning and growth that take place over months, years, and decades within medicine.^1^

The term “medical miracles” and the labeling of outcomes as such is an age‐old dilemma that is not without controversy. David Isaacs argued that calling spontaneous fortuitous occurrences in medicine “miracles” risks neglecting helpful scientific inquiry^3^ because it automatically renders the event inexplicable. Some suggest that believing in miracles encourages futile medical treatments, prolongs suffering, and forestalls appropriate transitions to palliative care. Since palliative care focuses on quality of life and providing relief from the symptoms of living with a serious illness, Rosoff reasoned that accepting palliative care involves accepting that not all patients can be cured. “Families and physicians who pursue miracles may deprive children of the benefits of palliative care.”^4^ Believing in miracles can be seen as giving families unrealistic expectations about what can be offered by medical teams or medicine in general. “Rarely do we acknowledge that modern systems consistently deliver at least 10% iatrogenic harm”—a number that Braithwaite reasoned has not changed despite numerous interventions and system‐wide attempts.^5^

### 1.1 Objectives

Whether miracles are seen as a source of conflict between healthcare teams and families, a foolish or even harmful belief, or a hindrance to system‐wide improvements and scientific inquiry, miracles within medicine have long been questioned. We were interested in the perspectives of pediatricians on miracles and how that has informed their care of patients and families. In addition, we sought to explore the intersection between the medical sciences and faith and how we should think about miracles through narrative inquiry as a research methodology.

## 2 RESEARCH DESIGN AND METHODS

Narrative inquiry invites readers to consider the larger social, familial, and institutional narratives which help shape a person's experiences. We conducted interviews and collected stories from physicians who work directly in pediatric clinical care. As selection criteria, we used purposeful sampling; we emailed seven pediatricians in the Department of Pediatrics at the University of Alberta in Edmonton, Canada. We chose physicians we felt might provide us with rich stories about miracles they have experienced in their clinical practice. Four pediatricians agreed to participate who had backgrounds in pediatric infectious diseases, neurodevelopment, intensive care, and hepatology. Three declined. One potential participant explained that they did not believe in miracles, while the others had unknown reasons.

### 2.1 Data collection

We used several sample interview questions to begin the conversations. However, the follow‐up questions were spontaneous and mainly guided by what the participant shared.

### 2.2 Data analysis

Throughout the course of the inquiry, we took notes during the interviews and transcribed all of the interviews using transcription software.^6^ We paid particular attention to the chronology of events and scheduled follow‐up conversations with the collaborators to explore important parts of a story or to ask about their thoughts on a question or thread that emerged. The specific number and length of the conversations varied among individuals depending on their level of comfort, availability, and depth of the conversation. Interviews were analyzed individually and as a group and after multiple discussions, we identified two common threads.

## 3 RESULTS

First, the word miracle can be used in a variety of ways, but participants described “miracles” as fortuitous events that invoked a sense of wonder due to contextual factors such as geography, resource availability, time in history but also previous experiences and the expectations of the clinician. Second, there is a complex dynamic between the medical sciences and the faith commitments of clinicians and families especially when facing life‐limiting or end‐of‐life illness.

### 3.1 Thread #1: Context Matters

Each pediatrician had a different working definition of what constitutes a miracle. The one commonality is that they all agreed that context matters. Factors such as geography, time in history, severity, and rarity of disease were all key components. Participants described the fear they felt when exposed to unfamiliar diseases, especially when practicing in other parts of the world. They described a “gap between what [they] were used to and what [they] saw.” Participant A was shocked when he witnessed a patient presenting with cerebral malaria and an altered level of consciousness. When Participant A visited Uganda for a global health project, his Ugandan colleagues who had seen many children with this presentation were far less alarmed than he was and anticipated a rapid recovery for this child. In rural Uganda, Participant A notes that physicians were much more frightened by jaundice, routinely seen by Canadian pediatricians, because they were unable to distinguish between benign and severe causes like biliary atresia due to the limited access to diagnostic imaging.

Several participants commented that the number of years a physician has been in practice also affects whether an event is labeled as a miracle. Participants theorized that younger trainees and physicians are more likely to be blinded by the infrastructure of medicine and have a greater tendency to “feel invincible” and take credit for a patient's success. Several participants noted that in resource‐poor areas, improbable and dramatic recoveries were more difficult to explain. In more affluent countries, participants noted that young physicians are more likely to attribute a patient's recovery to the medical team, technology, and resources. Participants shared that over their careers and with years of experience, especially with medicine in resource‐limited settings, they now have a greater appreciation for the unpredictability and uncertainty of medicine.

### 3.2 The Story of Sam

Perhaps the best examples of medical uncertainty and unpredictability are the stories of miraculous events. Participants B and C in separate interviews talked about the same patient and described him as a Christmas miracle. Sam's family woke one morning to discover that his diaper was full of dark red blood. They went to the emergency department for the first time, where his doctors eventually discovered an abdominal teratoma. Sam was a tiny 11‐month‐old with a mass the size of grapefruit in his belly. He was initially operated on at a different center in a neighboring city. While his surgeons were trying to resect the benign tumor, the blood supply to his superior mesenteric and celiac arteries was inadvertently compromised, resulting in complete small bowel and liver ischemia, which soon progressed to septic shock. Sam was transferred to another center for a multiorgan transplant.

On arrival to the intensive care, the team spoke to the family about their goals of care and shared that without an organ donor, Sam likely had between 12 and 24 h left to live. However, Sam continued to hold on. Over the next 8 weeks, he was on high inotropes, his bowels perforated, and he had a multiorgan failure. At one point, he was also on extracorporeal membrane oxygenation alert. However, somehow, his body was able to wall off the necrosed bowel and most of the liver. Right before Christmas, Sam received five new organs: a liver, small intestine, the bottom half of a stomach, a pancreas, and a partial colon. To the medical team's astonishment, after a highly extensive surgery with a history of poor outcomes, Sam recovered quickly and had very few complications posttransplant. Participants B and C both highlight Sam's and church community's faith and ardent prayers. No one could find a medical explanation for how he survived.

### 3.3 Wonder in the Ordinary and Everyday

Sam's story and the stories that are often associated with miracles tend to be dramatic accounts, which defy the odds and expectations of everyone involved. Participants A and C argued that miracles can also be seen in the small and ordinary things that can have a large impact. Participant C described one young teenager who required ongoing immunosuppressive therapy posttransplant and who also suffered from depression. There were many barriers that prevented this patient from taking the prescribed medications. However, after a summer of camp, building a better relationship with the team, and being surrounded by friends and peers with similar experiences, she began taking her medications regularly and attending all of her follow‐up appointments. As Participant C noted, “You begin to appreciate that those small things matter a lot, and probably cumulatively make a bigger difference to child health than the big time miracles that happen more occasionally.”

Participant A similarly celebrated the wonder in the ordinary and views the achievements of modern medicine as miracles. He noted that “we have become blasé about the tools in our tool belt” and that “our expectations have been colored by familiarity.” Participant A argued that forgetting that these scientific discoveries are miracles limits our curiosity and the process of scientific discovery. He argued that the “eureka discoveries are not as spontaneous as we think. But the way they are told appears spontaneous because it is the way narratives are naturally told. Science has always been tedious … Calling something a miracle does not mean we do not seek an explanation for why that outcome was so good.” Participants noted that there does not need to be a dichotomy between science and miracles.

Some participants attributed miraculous events to the inexplicable and to divine intervention. Others felt that some miracles have logical explanations that we have yet to discover. Participant B brought up the example of Francis Collins (former NIH director), who appeared on the cover of Time for his work heading the Human Genome Project and his book, The Language of God: A Scientist Presents Evidence for Belief. An overseer of the development of the SaRS‐CoV2 vaccines during the SaRS‐CoV2 pandemic, Collins was convinced that God would give a vaccine in record time, and he considered his prayers answered with the development of mRNA Covid vaccines. Although a more understood phenomenon and a catalyst for innovation, Collins considered the rapid development of mRNA Covid vaccines a miracle.

In response to the question, “does every miracle have an explainable natural phenomena?” Participant A responded, “perhaps more than we think.” Referencing his Christian background, Participant A argued that the world was created—and created with a finely tuned order and intelligent design that we are only beginning to uncover. He argued that miracles can be seen in defying impossible circumstances in ways beyond our current comprehension; however, we may have an explanation for some of these events in the future.

### 3.4 Thread #2: The Complex Dynamic between the Medical sciences, Spirituality, and Faith

We anticipated that families and physicians would have differing beliefs in miracles that could lead to conflict. Participant C shared about several rare instances where differing views on treatment between her medical team and the patient's family ended up involving child protective services. One of those cases involved a family that had strong religious beliefs and fervently believed that their child would recover miraculously without the need for a transplant. Participant B similarly had a patient with diabetic ketoacidosis who became severely ill because the parents were praying that God would heal her without the need for insulin. Strongly held beliefs in medical miracles in these cases led to a rejection of modern medicine.

Likewise, Participant A also found that with the Ebola epidemic in the Democratic Republic of the Congo, “spirituality interfered with sound biomedical practices.” Ebola was a devastating disease. Patients suffered from hemorrhagic fever, hematemesis, hemoptysis, hematochezia, and bleeding from eyes. It was spread from monkeys to humans and then human to human and began in rural areas. Participant A explained that because many of their loved ones died and never returned, these communities became suspicious of urban medical teams, which included many foreign physicians. Set against the backdrop of war, conspiracy theories spread throughout rural communities. The prevailing story was that a witch cursed her two nephews with hemorrhagic fever because they had eaten her cat. Given these explanations for the origins of Ebola, Participant A explained that “it was difficult to convince people that there was a biomedical explanation such as a virus and that individuals and communities needed to take precautions.” The religious and cultural differences in worldviews around disease and illness became a large barrier to patients and their families trusting foreign medical teams and receiving treatment and care.^3^ participants shared these experiences of conflict between patients, families, and medical teams who held differing views on spirituality and Western medicine.

Unexpectedly, participants also shared their own *internal* conflicts between their beliefs in medical science and their faith commitments. Participant C described a hesitancy in labeling events as miracles, “Miracles tend to have a spiritual connotation … I think that there is a pervasive sentiment in medicine to be non‐spiritual and completely factual, logic and evidence‐based. So the use of the term miracle tends to be very restricted because of the spiritual connotation.” And yet, Participant C noted, “I think that people's spirituality plays a big role in their overall wellbeing. You come out of medical school and everything's about class of evidence … But there are other factors that influence well‐being. Health is not just the absence of disease.” Participants described that medicine is both objective science and yet subjective because it is patient‐centered.

Although participants had differing backgrounds and differing views on the role of religion and spirituality in patient care, they shared the belief that a patient's own religious beliefs needed to be acknowledged and respected. Participant B noted that “we, [as medical teams], have to realize that we can be secular and not believe in anything, but we cannot ignore that some patients do believe, and the patients do have their faith and that's what they grasp on to in times of despair. If you want to treat the families holistically, we have to respect that and we have to allow that.”

### 3.5 Facing death

A recurring question that arose was, “How do you hope for the best outcomes for your patients and even for a miracle while facing death and basing your prognosis on current evidence?” Participant D answered the question by describing her personal experience being in the hospital at her father's bedside. “My father had a motto, ‘We are going to hope for the best and plan for the worst.’ We are praying for a miracle, we are looking and expecting now but we're still going to do our part … we're still going to fight … and if God intercedes … then we will be overjoyed. But if He doesn't, we're still going to know that He's still in those moments. We just don't know how they all connect.” Participant D described how her understanding of suffering and faith shifted and changed during that time. Although her father was sick and in the hospital, it was also a treasured time to be at his bedside and to spend uninterrupted time with him in a way they had not before. A miracle did not occur with a spontaneous cure or recovery for her father, but their time together was an unexpected providence that deepened their relationship.

Several participants critiqued their own society in North America and how it differs from other parts of the world. “If you look at the commercials on TV, you will see that a lot of commercials are about pain and painkillers. The issue is that our society doesn't like pain and cannot see pain as a virtue, as something that you can learn from. [Death here] is always seen as tragic and to be avoided. However, in South America, for example, there is the Day of the Dead … You go to the cemetery, you bring flowers, and there's a big party … [You] celebrate the dead.” (Participant B). Participant B argued that our current medical community's view of death and medicine needs to change.

Language such as “medicine has reached its limits” perpetuates a kind of thinking that medicine is simply about cures, recoveries, and treatments. Participant C contrasted the rooms of palliative care patients with code blues. Their rooms are quiet and the medical team tends to scatter and families can be left feeling abandoned. She noted that medical teams in general often shy away from difficult conversations and reserve these parts of care for palliative care teams, social workers, or hospital chaplains. Participant B argued that physicians, “often push and hope for a miracle, because we cannot accept that death is coming and we view death as defeat … [we] prolong suffering, because we're not prepared to let go. Not [just] the family, but many times it's us [the medical team]. We keep thinking that we can do another surgery or another treatment when we know that it is not going to work. [This is] called dysthanasia, “a bad death".” Several participants alluded to orthothanasia or “a good death” and letting go when further interventions were doing more harm than good.

## 4 DISCUSSION

Our study sought to explore some of the experiences of miracles in pediatric medicine. We identified two threads woven throughout the interviews. First, the word *miracle* can be used in a variety of ways but participants described *miracles* mostly as favorable and fortuitous events that invoked a sense of wonder due to contextual factors such as geography, resource availability, time in history but also previous experiences and the expectations of the clinician. Second, there is a complex dynamic between the medical sciences and the faith commitments that clinicians and families bring to situations of treatment and dying.

### 4.1 Definitions

The difficulty with the word “miracle” is that it can be used in a variety of ways and is interpreted as such depending on the context. Webster's 1913 dictionary defines a miracle as a “wonder or wonderful thing” or as “an event contrary to the established course of things.”^2^ Not only are contextual factors such as geography, access to resources, and time in history components in determining a miracle as such, but previous experiences and the expectations of the clinician also have a large impact.

Participants theorized that with more experience, they now appreciate the limits of medical technology and recognize the unpredictability and uncertainty of medicine more, describing how identical treatments and steps that follow the current guidelines can have drastically different outcomes in different patients. Much like Braithwaite's arguments, not only is medicine full of uncertainty and unpredictability but patients are regularly exposed to iatrogenic harm.^5^ In the case of Sam, what was initially a benign tumor became a life‐threatening issue due to the accidental ligation of major blood vessels during surgery. With this understanding, participants argued that we should have a greater appreciation and receptiveness to the favorable and fortuitous events that occur whether through medical technology or in spite of medical error.

Although the Latin word for *miracle* means wonder or marvel,^1^ the Greek word for *miracle* is “simaios” meaning a “sign.”^7^ Whether miracles are seen in dramatic events like the story of Sam or in the use of artesunate for cerebral malaria,^2^ participants expressed awe that perhaps all of these events point to workings beyond human agents and infrastructure. Participants emphasized celebrating the ordinary and everyday medical achievements and argued that perhaps miracles need not be inexplicable or rare occurrences.

### 4.2 Faith and the Medical Sciences

Albert Einstein once said, “Everyone who is seriously involved in the pursuit of science becomes convinced that a spirit is manifest in the laws of the Universe—a Spirit vastly superior to that of man and one in the face of which we with our modest powers must feel humble. In this way, the pursuit of science leads to a religious feeling of a special sort, which is indeed quite different from the religiosity of someone more naïve.”^7^ As one of the most prominent scientists, Einstein himself spoke of the limits of science. “Medicine is both an objective science and yet subjective because it is patient‐centred. We can be scientific and as detached from spirituality as much as you want, but when you have a patient encounter, and the patients are living in front of you, you cannot ignore it” (Participant C). Several participants shared the difficulty with staying objective as physicians when confronted with death and dying. Participants talked about the humanity they experienced when witnessing a family lose a child but also when faced with their own family members falling ill.

Isaacs believed that miracles and the medical sciences are incompatible.^3^ Using case reports documenting the spontaneous clearance of HIV, he argued that calling such spontaneous occurrences “miracles” risks neglecting helpful scientific inquiry^3^ because it automatically renders the event incomprehensible. Several participants disagreed and argued instead that believing in miracles encourages scientific exploration and discovery. Participant A alluded to arguments around our finely tuned universe. Believing in an intelligent design, he argued that our world can be understood and that belief is a large motivator in his research. To him and other participants, unbelief in miracles limits our curiosity.

Participant B brought up the example of Francis Collins and marveling at the mRNA Covid vaccines. Although not inexplicable, the rapid development, collaboration between researchers, time in history, and present and future potential impact are extroadinary—perhaps even miraculous. As we explored the dynamic between the medical sciences and faith, we ventured beyond the borders of science. Participants concluded that there must be a place for both rational scientific inquiry but also warned against rationalist hubris. Pope John Paul II wrote, “Science can purify religion from error and superstition; religion can purify science from idolatry and false absolutes. Each can draw the other into a wider world, a world in which both can flourish.”^8^

### 4.3 Miracles and palliative care

Our conversations around miracles brought into question what it means to be a good physician, especially during end‐of‐life care. Rosoff reasoned that accepting and transitioning to palliative care involves accepting that not all patients can be cured.^4^ He argued that pursuing a miracle through heroic measures can be more harmful to the family. It can be seen as giving families unrealistic expectations about what medical teams, or medicine in general, can offer.

Several participants shared that our views on medicine and palliative care need to shift. Participants noted that palliative care does not necessarily begin as soon as the prognosis is declared. Rather all throughout the patient's care, we should be striving to relieve pain and provide comfort. In regard to the end‐of‐life stages of care, however, participants shared that death is not admitting defeat or failure. From a personal perspective, Participant C described her own journey with her father during his time in the hospital. From a cultural perspective, Participant B described the Mexican holiday, Día de los Muertos (“Day of the Dead”), celebrating previous ancestors. Sharing personal and stories in practice of when the hoped for miracle does not occur, participants noted that the best outcome for patients does not always look like believing in a dramatic miracle or performing heroic measures but finding ways to preserve the dignity and comfort of the patient and celebrate their life and impact.

For Sam to receive five organs and for his miracle to take place, another family had to suffer a tragic loss. Some might conclude that medicine failed the family that lost their child or that medicine reached its limits for the patient who was Sam's organ donor. However, several participants noted that an important part of medicine is facing even the most difficult of prognoses with families. Preparing for death is a way of caring well for patients and their loved ones. Participants strongly believed that medicine should never be just about the treatments and cures. What hope may look like for a family may evolve over time as the clinical picture grows more clear. Being a good physician means not shying away from our own humanity and the humanity of patients. Participants shared their own personal experiences as medical professionals but also as parents, sons, and daughters. Hope may mean praying for a miraculous cure, but for another family, hope may mean finding purpose in the suffering. When patients are dying, it is the role of the physician to do everything possible to ensure they have, as Participant B describes, a “good death.” Participants B and D both highlighted in their experiences that comfort measures and being present for families at the bedside during end‐of‐life care are roles not only reserved for social workers or spiritual chaplains but also for physicians.

Even if one may not believe in miracles, participants noted a responsibility to leave space for the patient's spiritual and religious beliefs. Patient‐centered care means trying to understand a patient and their family's beliefs and values no matter how different or foreign they may be while keeping the patient's best interest in mind. These values held in tension may occasionally lead to both discordance within the healthcare provider themselves as well as between medical teams and families.

### 4.4 Limitations

One of the main limitations or challenges with the word “miracle” is that it has such a broad scope. The description of medical discoveries as miracles raises significantly different issues than “clinical” miracles in the sense of unexpected cures. Clinical miracles can further be subdivided as events against any reasonable prediction and without any explanation—and the marvels present in modern medicine that have an explanation but are wonders that should not be taken for granted. Others may argue that miracles can also be seen in the ordinary and everyday wonders of human life. The challenge of this topic is that it raises questions in so many different directions and the definition of miracles was so varied among our participants that it is difficult to capture with adequate depth.

## 5 CONCLUSION

Narrative inquiry provides a structure in which to ask questions. Through the retelling of stories and our discussions around miracles, questions emerged about what is a miracle, how should we view them, and how do miracles affect scientific exploration and patient care? Sam and Joshua Bell's stories are rare and extraordinary occurrences. But participants also expressed an appreciation for the seemingly ordinary tools in healthcare and everyday events in the lives of patients. Exploring the topic of medical miracles allowed us to better understand the social discourses that shape the perceptions of miracles, death, and the role of the physician. These stories of miracles—big and small—offer us hope and possibility in a time when we as a medical community may have lost our sense of wonder and even the ability to notice the miracles around us.

## AUTHOR CONTRIBUTIONS

**Geraldine Huynh**: Conceptualization; data curation; formal analysis; funding acquisition; investigation; methodology; project administration; writing—original draft; writing—review and editing. **Marghalara Rashid**: Conceptualization; data curation; formal analysis; funding acquisition; investigation; methodology; project administration; supervision; writing—original draft; writing—review and editing. **Jessica L. Foulds**: Conceptualization; data curation; formal analysis; funding acquisition; investigation; methodology; project administration; supervision; writing—original draft; writing—review and editing. All authors have read and approved the final version of the manuscript. Geraldine Huynh had full access to all of the data in this study and takes complete responsibility for the integrity of the data and the accuracy of the data analysis.

## CONFLICT OF INTEREST STATEMENT

The authors declare no conflict of interest.

## ETHICS STATEMENT

Informed consent was obtained for experimentation with human subjects and ethics was obtained (Pro00106628). Certain details of the patient stories have been changed to protect their confidentiality.

## TRANSPARENCY STATEMENT

The lead author Geraldine Huynh affirms that this manuscript is an honest, accurate, and transparent account of the study being reported; that no important aspects of the study have been omitted; and that any discrepancies from the study as planned (and, if relevant, registered) have been explained.

## Data Availability

The data that support the findings of this study are available on request from the corresponding author. The data are not publicly available due to privacy or ethical restrictions.
